# Supplementary material for: A quantitative model for the dynamics of target recognition and off-target rejection by the CRISPR-Cas Cascade complex
Source: Nat Commun. 2022 Dec 3;13:7460. doi: 10.1038/s41467-022-35116-5 (PMC9718816; doi:10.1038/s41467-022-35116-5)
Supplement: Supplementary file 1 — Supplementary Information [file 41467_2022_35116_MOESM1_ESM.pdf]

Supplementary Information

A quantitative model for the dynamics of target recognition and off-target rejection by the CRISPR-Cas Cascade complex

Marius Rutkauskas[1], Inga Songailiene[2], Patrick Irmisch[1], Felix E. Kemmerich[1], Tomas Sinkunas[2], Virginijus Siksnys[2], Ralf Seidel[1]

[1]Peter Debye Institute for Soft Matter Physics, Universität Leipzig, 04103 Leipzig, Germany

[2]Institute of Biotechnology, Life Sciences Center, Vilnius University, Saulėtekis ave. 7, Vilnius 10257, Lithuania

Supplementary Note 1: Calculation of first passage times between different states of a one-dimensional random walk

A) First passage time for terminal positions of start and end state

As described in the main text, we consider a one-dimensional (linear) random walk process containing $N+1$ positions $(0,1,2,\ldots,N)$. The model shall be fully parametrized by a set of rate constants $k_n^{+,-}$ describing the transitions at any position n to it's the two neighboring states (Supplementary Fig. 1). Rate constants marked with a '+' or a '-' correspond to forward and backward transitions, respectively (i.e. to transitions with increasing or decreasing position number).
We first consider a random walk that starts at position 0 and that terminates at position N which would correspond to full R-loop formation. The mean time T_{pass} that the walk needs to arrive for the first time at the last position is called mean first-passage time. To calculate T_{pass} we place a single particle into the system with p_n being the probability to find the particle at a given position n. We next introduce a reflecting boundary at the start position 0 (ensuring that there is no escape towards negative positions) and a transmissive boundary at position N. When a particle arrives at N, this boundary places the particle instantaneously to the start. Thus, the probability to find the particle at position N is zero at all times ($p_N = 0$).
The forward particle flux j_n between two neighboring positions n and $n+1$ is given by:

$$j_n = k_n^+ p_n - k_{n+1}^- p_{n+1},$$

We furthermore impose steady-state conditions for the system, i.e. the probabilities p_n shall not change over time:

$$0 = \frac{dp_n}{dt} = j_{n-1} - j_n$$

From this condition we get that the flux is constant throughout the system, i.e. $j_n = j_0$ for all n. For a single particle in the system, the mean first-passage time is then the reciprocal value of the steady-state particle flux j_0. With this the first equation can be transformed to:
Combining the upper two equations provides:

$$\frac{p_n}{j_0} = \frac{1}{k_n^+} + \frac{k_{n+1}^-}{k_n^+}\frac{p_{n+1}}{j_0}$$

With the boundary condition $p_N = 0$, we can obtain all ratios all p_n/j_0 can be obtained in a recursive manner:

$$\frac{p_{N-1}}{j_0} = \frac{1}{k_{N-1}^+},$$
$$\frac{p_{N-2}}{j_0} = \frac{1}{k_{N-2}^+} + \frac{1}{k_{N-2}^+}\frac{k_{N-1}^-}{k_{N-1}^+},$$
$$\frac{p_{N-3}}{j_0} = \frac{1}{k_{N-3}^+} + \frac{1}{k_{N-3}^+}\frac{k_{N-2}^-}{k_{N-2}^+} + \frac{1}{k_{N-3}^+}\frac{k_{N-2}^-}{k_{N-2}^+}\frac{k_{N-1}^-}{k_{N-1}^+},$$
$$\ldots.$$

Using the fact that the probability distribution p_n is normalized, the mean-first-passage time yields:

$$\sum_{n=0}^{N}\frac{p_n}{j_0} = \frac{1}{j_0} = T_{\text{pass}}$$

and thus, a closed expression for the first passage time.

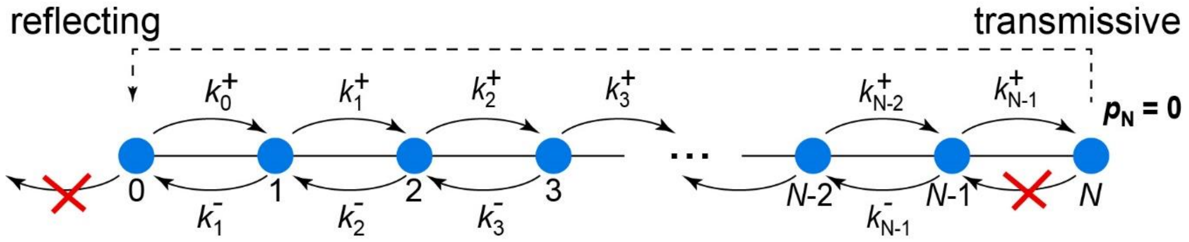

Supplementary Fig. 1 Scheme of the one-dimensional random walk with start at position 0 and end at position N. $k_n^{+,-}$ define the rate constants for the transitions between neighboring positions. To calculate the mean first-passage time for reaching position N, a reflective boundary is introduced at position 0 while a transmissive boundary is placed at position N. A particle that reaches position N is instantaneously placed back to the start.

B) Transition rates for a bidirectional random walk from an internal start to end states at either side

Longer lived R-loop intermediates that form in front of single mismatch positions can either collapse (return to position 0) or expand to form a full R-loop state (reach position N). In the framework of a one-dimensional random walk this can be described by a bidirectional walk that starts at an internal position m and that has two possible end states. To calculate mean transition rates to either end state, we place a single particle together with transmissive boundaries at either end into the system and impose steady-state conditions (Supplementary Fig. 2). The particle flux from the start position m splits into a flux j_- towards 0 and a flux j_+ towards N. Due to steady state conditions (see above), $j_- = const.$ between any two adjacent positions smaller than or equal to m and similarly $j_+ = const.$ between any two adjacent positions larger than or equal to m. With:

$$j_n = k_n^+ p_n - k_{n+1}^- p_{n+1}$$

and $p_0 = 0$ as well as $p_N = 0$ (due to the transmissive boundaries), we can obtain all ratios all p_n normalized by the respective flux in a recursive manner.

For positions larger than m we get as before:

$$\frac{p_{N-1}}{j_+} = \frac{1}{k_{N-1}^+},$$

$$\frac{p_{N-2}}{j_+} = \frac{1}{k_{N-2}^+} + \frac{1}{k_{N-2}^+}\frac{k_{N-1}^-}{k_{N-1}^+},$$

....

For positions smaller than m we get analogously:

$$\frac{p_1}{j_-} = \frac{1}{k_1^-},$$

$$\frac{p_2}{j_-} = \frac{1}{k_2^-} + \frac{1}{k_2^-}\frac{k_1^+}{k_1^-},$$

....

We finally obtain expressions for p_m/j_- and p_m/j_+. The ratio between the two values:

$$r = \frac{p_m}{j_-} \Big/ \frac{p_m}{j_+} = \frac{j_+}{j_-}$$

Provides the ratio between forward and backwards flux, i.e. the ratio that a particle reaches the end at N vs. the end at 0. Let $j = j_+ + j_-$ be the total particle flux in the system. We can then express backward and forward flux as:

$$j_- = (j_+ + j_-)\frac{j_-}{j_+ + j_-} = j\frac{1}{1+r} = k_-$$

and

$$j_+ = (j_+ + j_-)\frac{j_+}{j_+ + j_-} = j\frac{1}{1+1/r} = k_+$$

j_+ and j_- correspond to the unidirectional particle flux at which particles arrive at either end. They correspond thus to the transition rates k_- and k_+ from position m to position 0 and N, respectively, as denoted in the equations above.

With this we obtain expressions for the particle probabilities normalized by the total flux p_n/j.

For $n \leq m$ we get:

$$\frac{p_n}{j} = \frac{p_n}{j_-}(1+r)$$

and for $n \geq m$ we get:

$$\frac{p_n}{j} = \frac{p_n}{j_+}(1+1/r)$$

Using the normalization condition that only one particle is in the system at all times we get an expression for the total flux:

$$\sum_{n=0}^{N} \frac{p_n}{j} = \frac{1}{j}$$

With this the transition rates k_+ and k_- can be finally calculated.
Please note that this approach allows to calculate any possible rates and probabilities to make a transition to a given state in the system. When considering more intermediate states (e.g. due to more mismatches present), the two end state positions 0 and N need to be correspondingly replaced by the positions of the actual states that are adjacent to the start position. Furthermore, the probabilities to make it either towards the end in forward or backward direction are given as:

$$p_+ = \frac{j_+}{j} = \frac{1}{1 + 1/r}$$

and

$$p_- = \frac{j_-}{j} = \frac{1}{1 + r}$$

Setting $m = 1$ allows then also to calculate the probability that following PAM binding a full R-loop is formed, i.e. the target would be recognized.

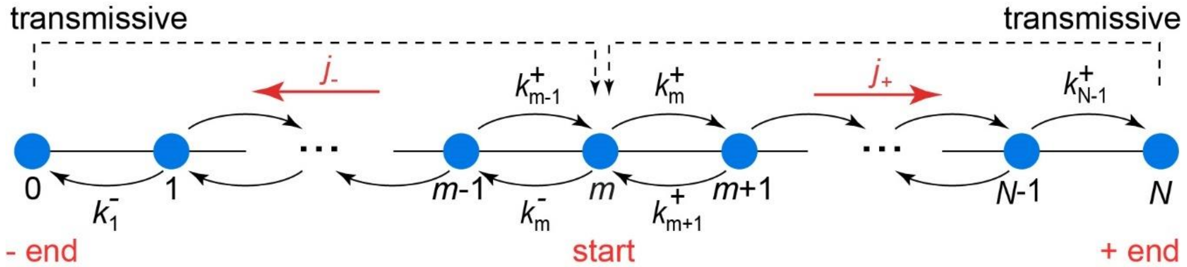

Supplementary Fig. 2 Scheme of a one-dimensional random walk with start at an internal position *m* and two possible end states at either side. $k_n^{+,-}$ define the rate constants for the transitions between neighboring positions. To calculate the mean rates for reaching either end at positions 0 and N, transmissive boundary conditions are placed at position at both ends. A particle that reaches an end is instantaneously placed back to the start. In steady state there are two different particle fluxes: j_- and j_+ for particles that traveled to 0 and N, respectively.

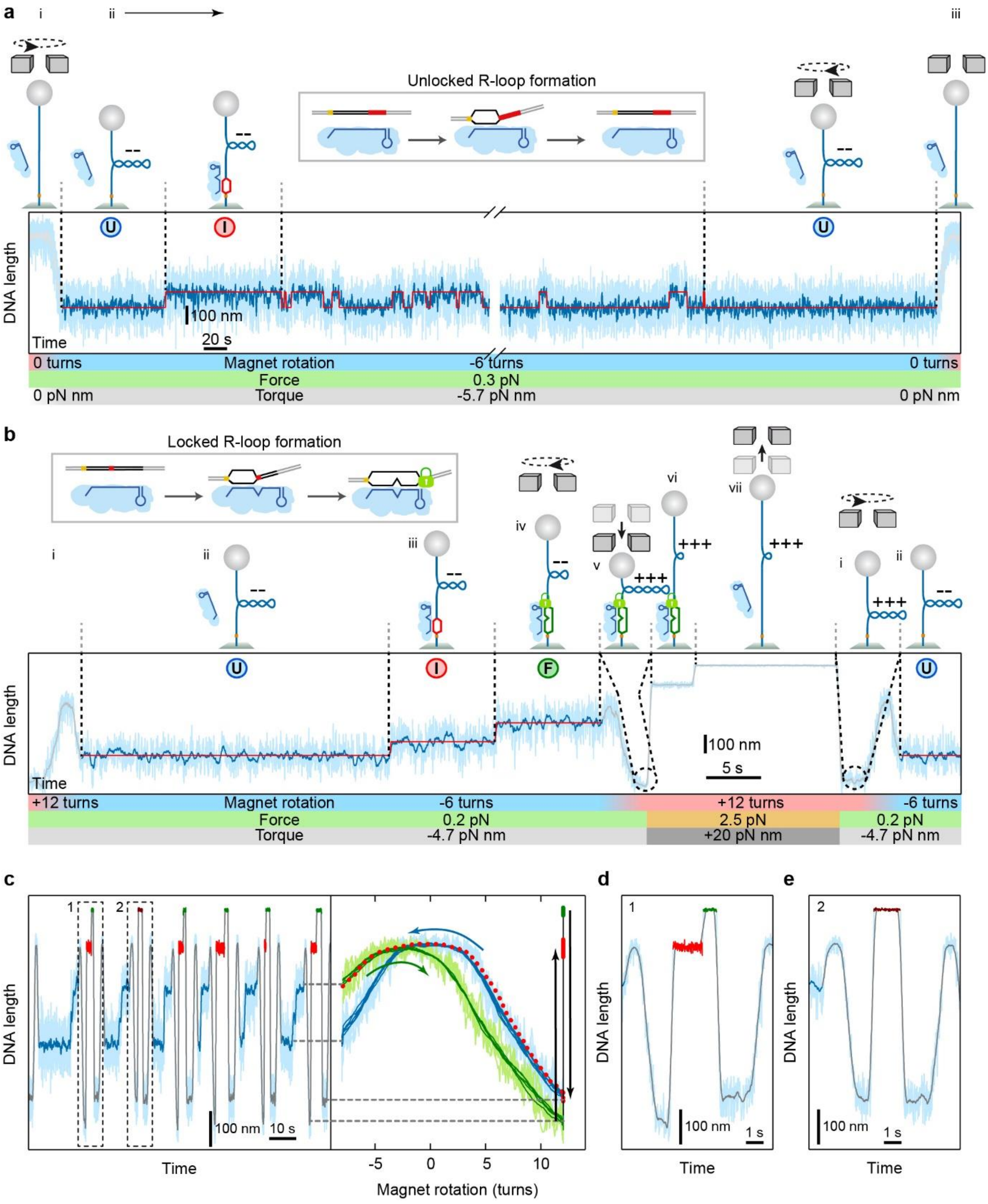

Supplementary Fig. 3 Detailed representation of DNA length, applied turns, force and torque during the different R-loop formation experiments using magnetic tweezers. **a** Experiment to study the R-loop dynamics on a target that does not lock due to ≥6 bp PAM distal mutations (20 bp for the depicted example or 12 matching bp). The experiment starts with supercoiling the DNA molecule from 0 turns to -3 to -8 negative turns at constant magnet position (i). The molecule length reduces due to the DNA writhe. The magnetic field force is kept constant providing a constant negative torque acting on the DNA that is controlled by the force (ii). Thermally driven R-loop formation and collapse events seen as discrete changes of the DNA length (see two state approximation shown as red line as well as cartoons on top) are followed over a sufficiently long time. Finally, negative DNA supercoiling is removed by

turning the magnets to the initial 0 turns leading to a DNA length increase (iii). The light blue trajectory represents the raw DNA length data collected at 120 Hz, the dark blue trajectory the DNA length after sliding-window filtering to 7.5 Hz. Red lines represent the two-state approximation of the trajectory. **b** Experiment to study the R-loop formation kinetics in case of locking. In order to measure multiple R-loop formation events Cascade needs to be dissociated from the locked state. First, R-loop formation is facilitated by applying negative turns at low force of 0.1 – 0.45 pN (transition from i to ii). After observing formation of I state (iii) and subsequent locked R-loop formation (seen as a sufficient DNA length increase corresponding to the F state iv), the R-loop dissociation is induced by supercoiling the DNA towards positive turns (v) followed by the application of a higher force of ~2.5 pN (vi) that provides an increased positive torque. R-loop dissociation is observed as a discrete DNA length increase (vii). For a new R-loop formation experiment, the force is again lowered and the DNA is supercoiled to the initial negative turns (i and ii). **c** Example of multiple R-loop formation-dissociation cycles for a substrate containing a mismatch at position 7. Left panel represents the DNA length time trajectory depicted as in (**b**). Red areas of the trajectory represent R-loop at high force and positive supercoiling as vi in (**b**). Green areas of the trajectory represent DNA length after the R-loop dissociation as vii in (**b**). Right panel represents DNA length dependency on magnet rotation. Blue curve shows magnet rotation after R-loop dissociation from positive supercoiling to negative (i in (**b**)). After R-loop formation (seen as an abrupt jump of DNA length in left panel or transition from ii to iii to iv in (**b**)) DNA is being supercoiled from negative turns to positive turns and is shown as a green curve (transition from iv to v in (**b**)). In some rare cases the full R-loop was formed but remained unlocked (depicted as brown area in left panel). In this case the R-loop collapse occurred around zero turns where it could not be seen (red dashed curve in the right panel). These events were not considered in further data analysis. To verify whether the R-loop was locked, we monitored the expected sudden DNA length increase upon R-loop dissociation as well as the shift of the rotation curves at positive turns expected for stable locked R-loops.
d, **e** Enlarged views of the dissociation trajectories from (**c**). In D R-loop presence is observed as lower magnetic bead position after DNA is positively supercoiled (v in (**b**)) and as a presence of two distinct states after the increase of force (red and green areas, vi and vii in (**b**)). In case of R-loop dissociation during transition through 0 turns (red dashed curve in right panel of (**c**)) magnetic bead does not go as low as in the presence of R-loop (i in (**b**)) and only one state is observed after the increase of force (brown area in left panel of (**c**), vii in (**b**)) as represented in (**e**).

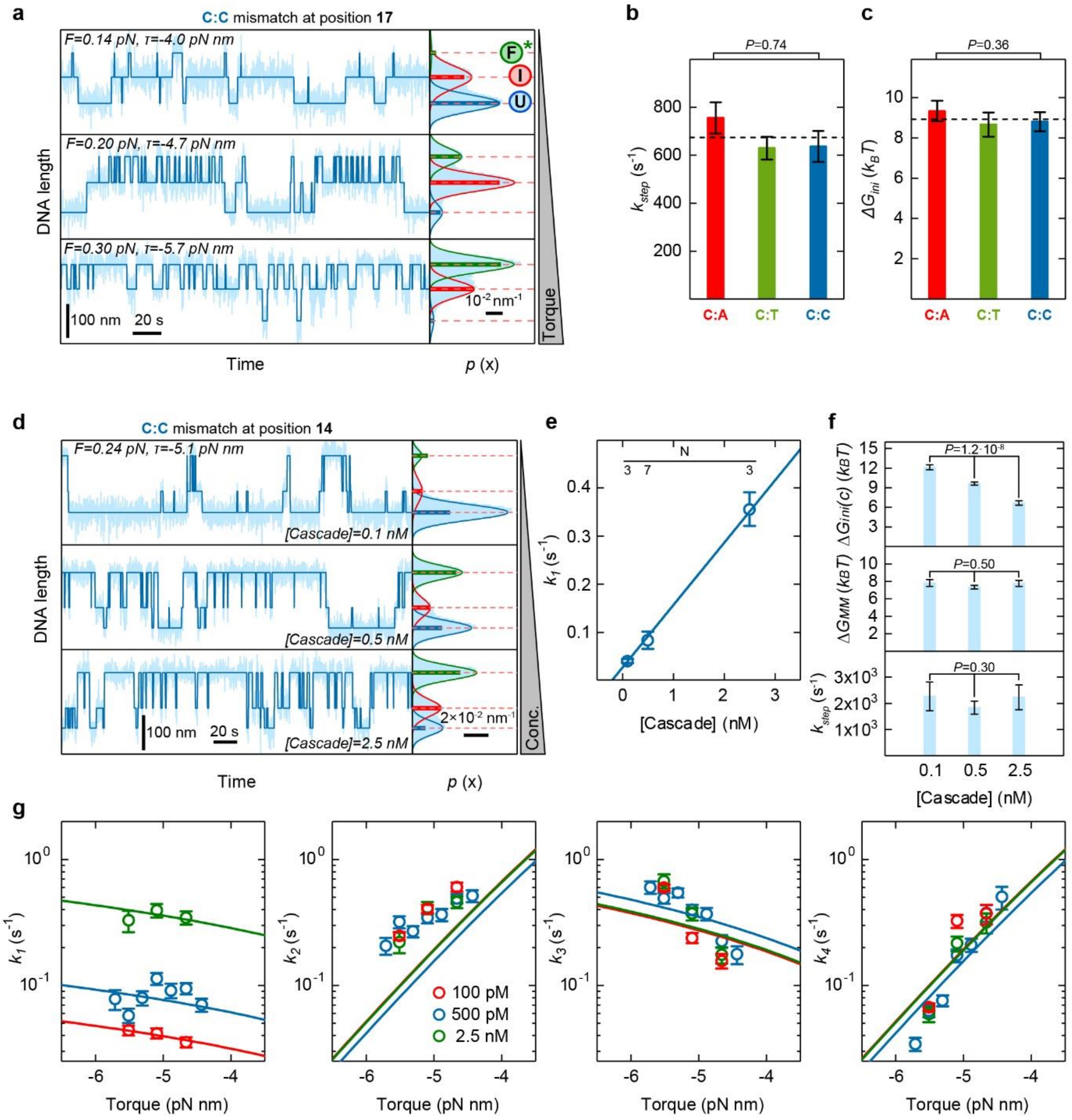

Supplementary Fig. 4 R-loop dynamics in presence of a single mismatch measured at different concentrations. a Trajectories and histograms of the DNA length recorded for different applied torques using a target with C:C mismatch at position 17 (light blue). Solid lines in the histograms represent Gaussian fits to the 3 different states, while horizontal dashed lines indicate the average DNA length of each state. Bars represent theoretically predicted occupancies using the parameters shown in Supplementary Table 1. **b** Single base-pair stepping rates for the different mismatches obtained from the transition rate fits. The dashed line represents the mean rate. Error bars correspond to SD of the fit parameter (67% confidence interval). **c** Free energy change of R-loop initiation at a Cascade concentration of 170 nM for the different mismatches obtained from the transition rate fits. The dashed line represents the mean. Error bars correspond to SD of the fit parameter (67% confidence interval). **d** DNA length trajectories and occupancies measured at different Cascade concentrations on a target containing a C:C mismatch at position 14 and 6 PAM-distal mismatches. With increasing concentrations the full R-loop state F^* becomes increasingly populated. **e** Intermediate R-loop formation rate k_1 as a function of the Cascade concentration at -4.7 pN nm (open circles). The blue line represents a linear fit to the data. Error bars

correspond to SEM. **f** Best fit parameters obtained for the different concentrations from the fits of the transition rates between the different R-loop intermediates (see (**g**)). Error bars correspond to SD of the fit parameter (67% confidence interval). **g** Experimental transition rates as a function of torque for the different Cascade concentrations (open circles). Global fits to all rates at the given concentrations are shown as solid lines. Error bars correspond to SEM. Precise sample sizes are given in the Supplementary Table 6. For statistical testing one-way ANOVA was used.

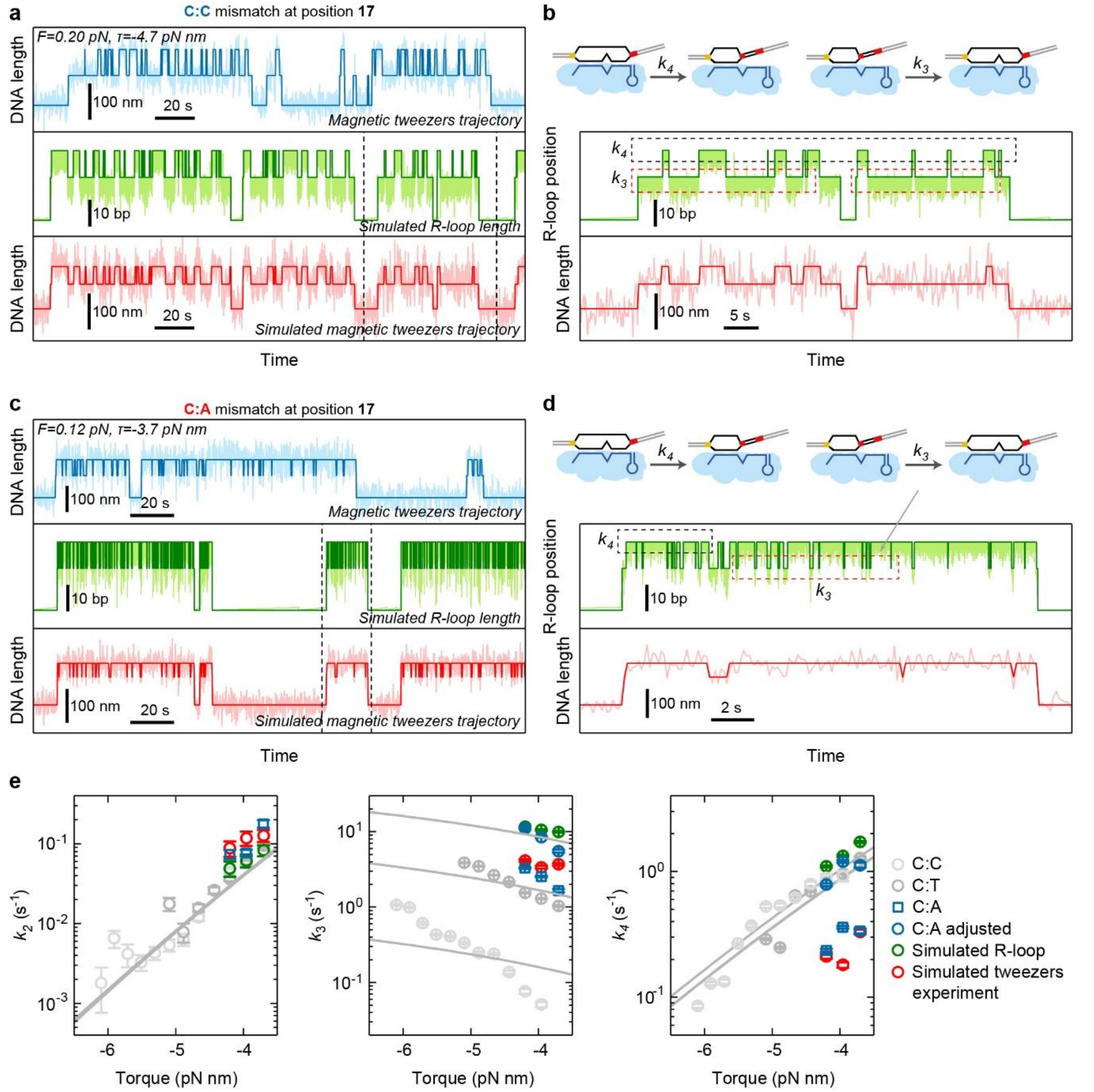

Supplementary Fig. 5 Simulations of R-loop length and magnetic tweezers experiments for single mismatch targets. a, **c** Comparison between trajectories of a magnetic tweezers experiment (blue), a random walk simulation of the R-loop length (green) and a Brownian dynamics simulation of a magnetic tweezers experiment (red) for a target containing C:C mismatch (**a**) and C:A mismatch (**c**) at the position 17 and 6 terminal mismatches. Light colors show the raw results of the experiments and simulations while dark colors show corresponding 3-state approximations. **b**, **d** Enlarged view into the simulated trajectories from areas separated by dashed lines in (**a**) and (**c**) correspondingly depicting transitions extracted by the 3-state approximations of the simulated magnetic tweezers trajectories. Dashed boxes depict transitions contributing to k_3 and k_4. **e** Comparison of the extracted rates for measured and simulated trajectories. The measured k_3 rate was adjusted by the ratio between measured k_4 rates for C:C, C:T and C:A mismatches. Assuming k_4 for C:A mismatch is in the same range as for C:C and C:T, C:A values were shifted upward. The same ratio was used to shift k_3 values. After the adjustment, determined mismatch penalty values were used to perform Brownian dynamics simulations and to compare rates from the simulation experiment and magnetic tweezers experiment. Error bars in all plots correspond to SEM. Precise sample sizes are given in the Supplementary Table 6.

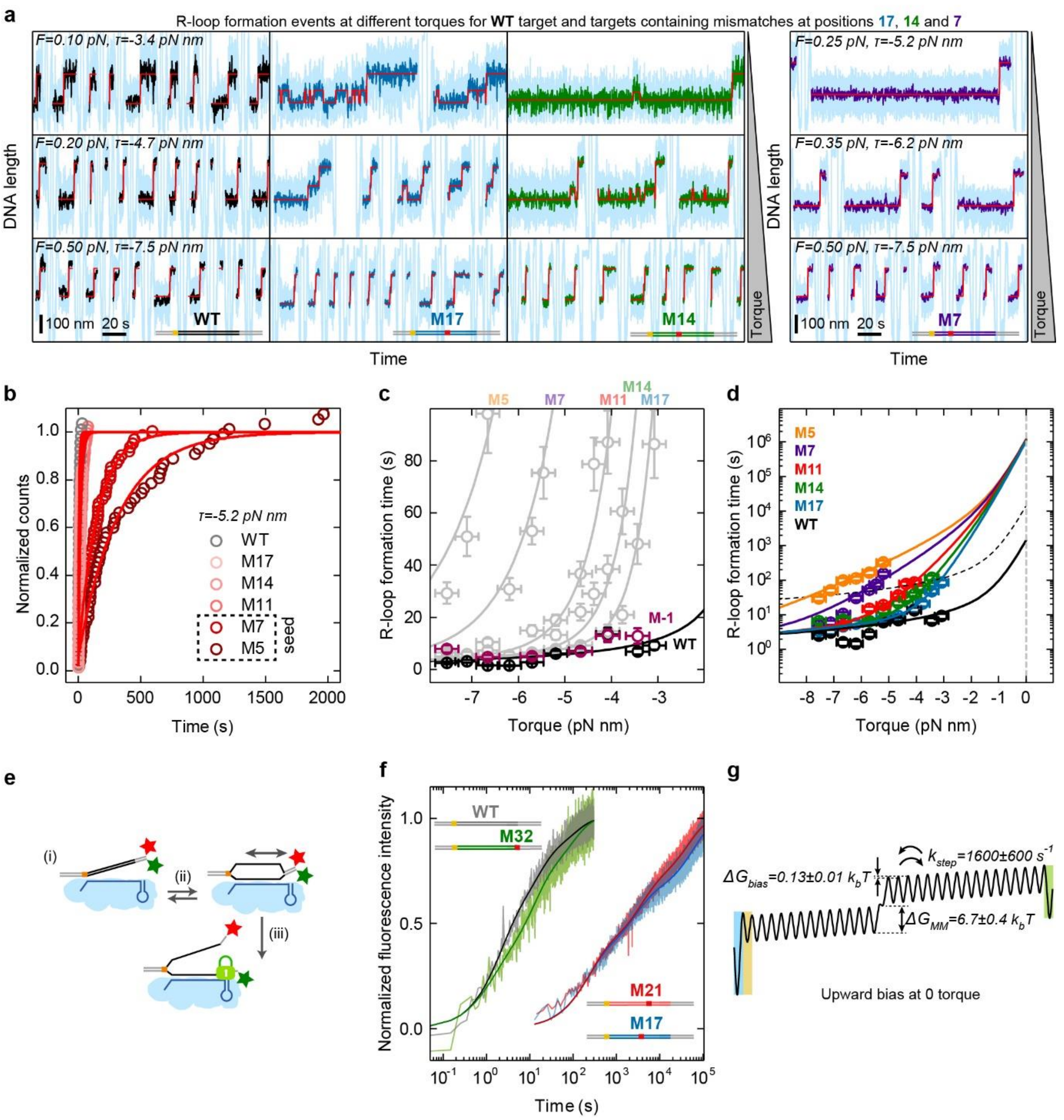

Supplementary Fig. 6 Additional data for locked R-loop formation on targets with single internal mismatches. a Example trajectories for R-loop formation on targets containing a single C:C mismatch at various positions as indicated). Colored sections represent the actual R-loop formation events. The width of a formation event is roughly proportional to the R-loop formation time, such that a visual impression of the R-loop formation time for the different mismatches and torques can be obtained. Light blue trajectories represent raw magnetic tweezers data collected at 120 Hz, dark trajectories represent the data after sliding-window filtering to 7.5 Hz, red lines represent 2-state approximations of the trajectories for WT and M7 targets (the intermediate state is too short-lived to be observed for M7) and 3-state approximations for M17 and M14 targets. **b** R-loop formation kinetics for the different targets at a torque of -5.2 pN nm (open circles) are represented as normalized event count over time of the event occurrence. Single exponential fits to the data are shown as solid lines. **c** Comparison of the torque dependence of R-loop formation for the WT target and a target with a PAM mutation at position -1 (see Supplementary Table 3). Data shown in gray is from Fig. 4 (main text). **d** Torque dependence of the R-loop formation times for the different targets with single internal mismatches plotted with a semi-logarithmic time scale including extrapolation

of the fit curves to zero torque. Noticeable, the difference between the different targets with single mismatches vanishes at zero torque, while a strong difference to the WT target persists. **e** Scheme of the fluorescence bulk solution measurements of the R-loop formation kinetics in absence of supercoiling (zero torque) involving a donor-acceptor dye pair at the PAM distal end of the target. The donor fluorescent signal increases mainly due to R-loop formation and locking (ii, iii) but not initial PAM binding (i). **f** Kinetics of R-loop formation in absence of supercoiling for WT and mismatched targets. All traces show the average of three replicates. **g** Depiction of the positively biased energy landscape of the R-loop formation for the target with the mismatch at position 15. Error bars in all plots correspond to SEM. Precise sample sizes are given in the Supplementary Table 6.

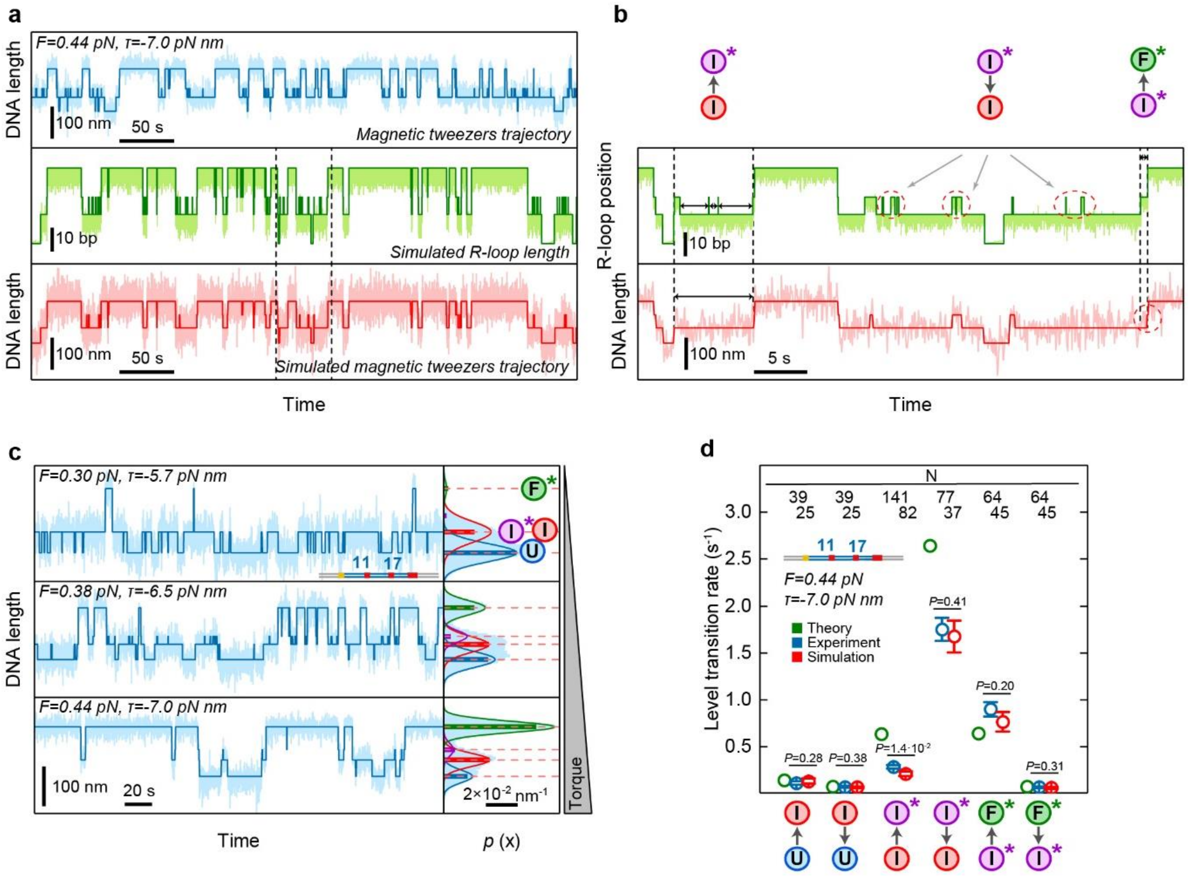

Supplementary Fig. 7 Simulations of R-loop length and magnetic tweezers experiments for double mismatch targets. a Comparison between trajectories of a magnetic tweezers experiment (blue), a random walk simulation of the R-loop length (green) and a Brownian dynamics simulation of a magnetic tweezers experiment (red) for a target containing C:C mismatches at the positions 11 and 17 and 6 terminal mismatches. Light colors show the raw results of the experiments and simulations while dark colors show corresponding 3-state approximations. **b** Enlarged view into the simulated trajectories from areas separated by dashed lines in (**a**) depicting transitions extracted by the 4-state approximations of the simulated magnetic tweezers trajectories. Dashed areas depict transitions that are affected by measurement limitations. **c** Trajectories and histograms of the DNA length recorded for different applied torques using a target with C:C mismatch at positions 11 and 17 (light blue). 4-state and 3-state (in case I and I* states were indistinguishable) approximations of the trajectories are shown as dark lines. Solid lines in the histograms represent Gaussian fits experimental histograms, while horizontal dashed lines indicate the average DNA length of each state. Bars represent theoretically predicted occupancies using the parameters shown in Supplementary Table 1. **d** Theoretical (green), experimental (blue) and simulation-based (red) transition rates between adjacent states for the 11-17 double-mismatch target at a torque of -7.0 pN nm. Error bars in all subplots correspond to SEM. For statistical testing one-tailed Z test was used.

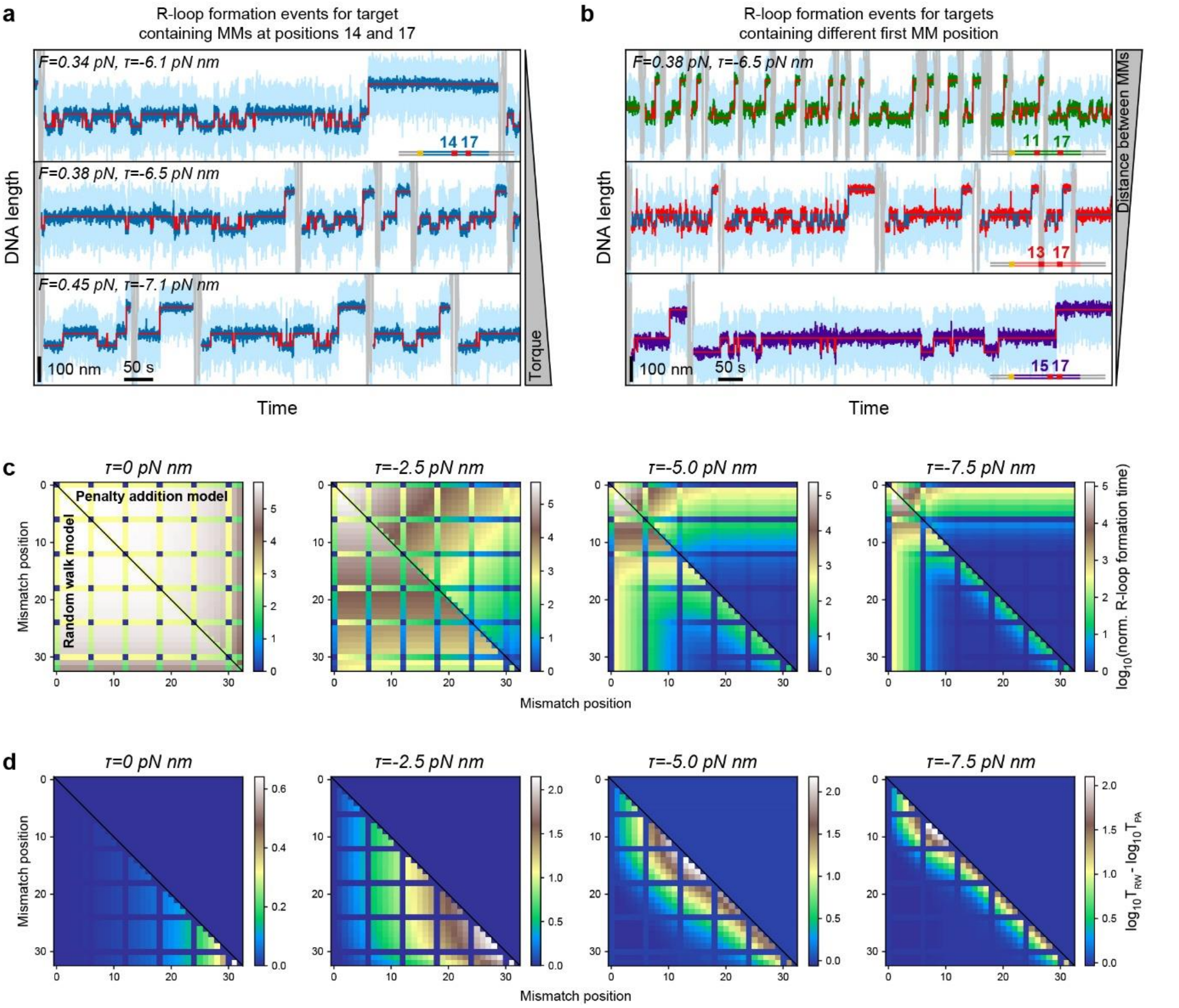

Supplementary Fig. 8 Impact of double mismatches on the R-loop formation times and transition rates between the states. a Successive R-loop formation events measured at different torques (as indicated) for mismatches at positions 14 and 17. Blue trajectory sections represent the actual R-loop formation events, while gray sections correspond to changes in the DNA supercoiling. The width of a formation event is roughly proportional to the R-loop formation time, such that a visual impression of the R-loop formation time for the different mismatches and torques can be obtained. Light blue trajectories represent raw DNA length data collected at 120 Hz, while dark trajectories are after data filtering to 3 Hz. **b** Successive R-loop formation events for mismatches at varying first mismatch positions; depicted as in (**a**). Blue trajectory represents the R-loop formation events for targets containing mismatches at positions 11 and 17, red – 13 and 17, violet – 15 and 17. **c** Normalized R-loop formation times for the different position combinations of double mismatches calculated for the random walk model (lower half) and for simple addition of energy penalties (upper half) at different torques. **d** Difference between the R-loop formation times of the random walk model (T_{RW}) and the simple penalty addition (T_{PA}) calculated by normalizing the plots in (**c**) by the results from simple penalty addition.

Supplementary Table 1. Best fit parameters for Figure 3 experiment.

Mismatch type	Mismatch position	k_{step} (s^{-1})	ΔG_{ini} (k_BT)	ΔG_{MM} (k_BT)	ΔG_{MM}*** *(DNA thermodyn.)* (k_BT)	ΔABA **** (k_BT)
C:C*	11	2000**	-	7.6±0.4	9.2	-
	13	3300±600	9.4±0.3	7.8±0.6	9.2	-
	14	1800±300	9.6±0.2	7.4±0.4	9.2	-
	15	4100±600	10.4±0.5	7.3±0.4	9.2	-
	16	2500±1200	10.8±1.6	8.1±0.6	9.2	-
	17	2100±100	11.2±0.5	8.0±0.2	9.2	-
C:C	17	640±70	8.8±0.5	6.7±0.3	10.5	4.5
C:T	17	630±50	8.7±0.6	4.3±0.2	8.3	3.7
C:A	17	760±70	9.3±0.5	2.7±0.1	7.5	3.2

***** Target sequences used here are designed to have the same neighboring bases and their sequences are presented in Supplementary Table 3.

****** k_{step} and ΔG_{MM} for C:C mismatch was obtained only from the fitting of k_3.

*** Determined for DNA:DNA duplex from nucleic acid thermodynamics using NUPAC [1].

******** Apparent binding energies of targets containing single mismatches from high throughput measurements[2]. Shown are average values of ΔABAs for all C:C, C:T and C:A mismatches in these experiments independent of the nearest neighbor bases. Please note that the ΔABAs were not corrected for position-dependent bias, such that they do not represent mismatch penalties according to the definition in our model.

Supplementary Table 2. Best fit parameters for Figure 3 experiments when including the prebias from Figure 4 experiments.

Mismatch type	Mismatch position	k_{step} (s^{-1})	ΔG_{MM} (k_BT)	ΔG_{ini} (k_BT)
C:C	17	230±20	5.2±0.2	7.5±0.5
C:T	17	260±20	2.9±0.1	7.5±0.6
C:A	17	340±30	1.0±0.2	8.3±0.5

Supplementary Table 3. Sequences of the oligonucleotides used in this study to produce target DNAs. PAM*** for Cascade is coloured yellow, matching part of the target sequence – green, mismatched bases of the target sequence – red, flipped out bases – grey.

DNA substrate	Sequences of dsDNA	Used in Figure	crRNA #
8 bp	5′-GACCACCCTTTTTGATATAATATACCTATTAGTTACCGGAGGGTGCGTATTCGGCAGATACGTTCTGAGGGAA<br>3′-CTGGTGGGAAAAACTATATTATATGGATAATCAATGGCCTCCCACGCATAAGCCGTCTATGCAAGACTCCCTT	2	1
10 bp	5′-GACCACCCTTTTTGATATAATATACCTATATGTTACCGGAGGGTGCGTATTCGGCAGATACGTTCTGAGGGAA<br>3′-CTGGTGGGAAAAACTATATTATATGGATATACAATGGCCTCCCACGCATAAGCCGTCTATGCAAGACTCCCTT	2	1
12 bp	5′-GACCACCCTTTTTGATATAATATACCTATATCATACCGGAGGGTGCGTATTCGGCAGATACGTTCTGAGGGAA<br>3′-CTGGTGGGAAAAACTATATTATATGGATATAGTATGGCCTCCCACGCATAAGCCGTCTATGCAAGACTCCCTT	2	1
14 bp	5′-GACCACCCTTTTTGATATAATATACCTATATCAATCCGGAGGGTGCGTATTCGGCAGATACGTTCTGAGGGAA<br>3′-CTGGTGGGAAAAACTATATTATATGGATATAGTTAGGCCTCCCACGCATAAGCCGTCTATGCAAGACTCCCTT	2	1
16 bp	5′-GACCACCCTTTTTGATATAATATACCTATATCAATGGGGAGGGTGCGTATTCGGCAGATACGTTCTGAGGGAA<br>3′-CTGGTGGGAAAAACTATATTATATGGATATAGTTACCCCTCCCACGCATAAGCCGTCTATGCAAGACTCCCTT	2	1
22 bp	5′-GACCACCCTTTTTGATATAATATACCTATATCAATGGCCTCCCTGCGTATTCGGCAGATACGTTCTGAGGGAA<br>3′-CTGGTGGGAAAAACTATATTATATGGATATAGTTACCGGAGGGACGCATAAGCCGTCTATGCAAGACTCCCTT	2	1
M17 C:C*	5′-GACCACCCTTTTTGATATAATATACCTATATCAATGGGCTCCCACGCTATTCGGCAGATACGTTCTGAGGGAA<br>3′-CTGGTGGGAAAAACTATATTATATGGATATAGTTACCCGAGGGTGCGATAAGCCGTCTATGCAAGACTCCCTT	3	1
M17 C:T*	5′-GACCACCCTTTTTGATATAATATACCTATATCAATGGACTCCCACGCTATTCGGCAGATACGTTCTGAGGGAA<br>3′-CTGGTGGGAAAAACTATATTATATGGATATAGTTACCTGAGGGTGCGATAAGCCGTCTATGCAAGACTCCCTT	3	1
M17 C:A*	5′-GACCACCCTTTTTGATATAATATACCTATATCAATGGTCTCCCACGCTATTCGGCAGATACGTTCTGAGGGAA<br>3′-CTGGTGGGAAAAACTATATTATATGGATATAGTTACCAGAGGGTGCGATAAGCCGTCTATGCAAGACTCCCTT	3	1
M11 C:C*	5′-GACCACCCTTTTTGATATAATATACTTATAGGGGCGGCGCGTGAGCGTATTCGGCAGATACGTTCTGAGGGAA<br>3′-CTGGTGGGAAAAACTATATTATATGAATATCCCCGCCGCGCACTCGCATAAGCCGTCTATGCAAGACTCCCTT	3	2
M13 C:C*	5′-GACCACCCTTTTTGATATAATATACTTATAGCGGGTGCGTGCGAGCGTATTCGGCAGATACGTTCTGAGGGAA<br>3′-CTGGTGGGAAAAACTATATTATATGAATATCGCCCACGCACGCTCGCATAAGCCGTCTATGCAAGACTCCCTT	3	3
M14 C:C*	5′-GACCACCCTTTTTGATATAATATACTTATAGCGGGGGCGCGTGAGCGTATTCGGCAGATACGTTCTGAGGGAA<br>3′-CTGGTGGGAAAAACTATATTATATGAATATCGCCCCCGCGCACTCGCATAAGCCGTCTATGCAAGACTCCCTT	3	2
M15 C:C*	5′-GACCACCCTTTTTGATATAATATACTTATAGCGAGGGCGTGCGAGCGTATTCGGCAGATACGTTCTGAGGGAA<br>3′-CTGGTGGGAAAAACTATATTATATGAATATCGCTCCCGCACGCTCGCATAAGCCGTCTATGCAAGACTCCCTT	3	4
M16 C:C*	5′-GACCACCCTTTTTGATATAATATACTTATAGCGATGGGGTGCGAGCGTATTCGGCAGATACGTTCTGAGGGAA<br>3′-CTGGTGGGAAAAACTATATTATATGAATATCGCTACCCCACGCTCGCATAAGCCGTCTATGCAAGACTCCCTT	3	5
M17 C:C*	5′-GACCACCCTTTTTGATATAATATACTTATAGCGAGCGGGTGCGAGCGTATTCGGCAGATACGTTCTGAGGGAA<br>3′-CTGGTGGGAAAAACTATATTATATGAATATCGCTCGCCCACGCTCGCATAAGCCGTCTATGCAAGACTCCCTT	3	2
WT	5′-GACCACCCTTTTTGATATAATATACCTATATCAATGGCCTCCCACGCATAAGCGCAGATACGTTCTGAGGGAA<br>3′-CTGGTGGGAAAAACTATATTATATGGATATAGTTACCGGAGGGTGCGTATTCGCGTCTATGCAAGACTCCCTT	4	1
M5	5′-GACCACCCTTTTTGATATAATAGCGGGATATCAATGCGCTCCCACGCATAAGCGCAGATACGTTCTGAGGGAA<br>3′-CTGGTGGGAAAAACTATATTATCGCCCTATAGTTACGCGAGGGTGCGTATTCGCGTCTATGCAAGACTCCCTT	4	6
M7	5′-GACCACCCTTTTTGATATAATATACCTTTATCAATGGCCTCCCACGCATAAGCGCAGATACGTTCTGAGGGAA<br>3′-CTGGTGGGAAAAACTATATTATATGGAAATAGTTACCGGAGGGTGCGTATTCGCGTCTATGCAAGACTCCCTT	4	1
M11	5′-GACCACCCTTTTTGATATAATATACTTATAGGGGCGGCGCGTGAGCGATAAGCGCAGATACGTTCTGAGGGAA<br>3′-CTGGTGGGAAAAACTATATTATATGAATATCCCCGCCGCGCACTCGCTATTCGCGTCTATGCAAGACTCCCTT	4	2
M14	5′-GACCACCCTTTTTGATATAATATACTTATAGCGGGGGCGCGTGAGCGATAAGCGCAGATACGTTCTGAGGGAA<br>3′-CTGGTGGGAAAAACTATATTATATGAATATCGCCCCCGCGCACTCGCTATTCGCGTCTATGCAAGACTCCCTT	4	2
M17	5′-GACCACCCTTTTTGATATAATATACTTATAGCGGCGGGGCGTGAGCGATAAGCGCAGATACGTTCTGAGGGAA<br>3′-CTGGTGGGAAAAACTATATTATATGAATATCGCCGCCCCGCACTCGCTATTCGCGTCTATGCAAGACTCCCTT	4	1
M21	5′-GACCACCCTTTTTGATATAATATACCTATATCAATGGCCTCGCACGCATAAGCGCAGATACGTTCTGAGGGAA<br>3′-CTGGTGGGAAAAACTATATTATATGGATATAGTTACCGGAGCGTGCGTATTCGCGTCTATGCAAGACTCCCTT	4	1
M11M17**	5′-GACCACCCTTTTTGATATAATATACTTATAGGGGCGGGGCGTGAGCGTATTCGGCAGATACGTTCTGAGGGAA<br>3′-CTGGTGGGAAAAACTATATTATATGAATATCCCCGCCCCGCACTCGCATAAGCCGTCTATGCAAGACTCCCTT	5	2
M13M17**	5′-GACCACCCTTTTTGATATAATATACTTATAGCGGGTGGGTGCGAGCGTATTCGGCAGATACGTTCTGAGGGAA<br>3′-CTGGTGGGAAAAACTATATTATATGAATATCGCCCACCCACGCTCGCATAAGCCGTCTATGCAAGACTCCCTT	5	3
M14M17**	5′-GACCACCCTTTTTGATATAATATACTTATAGCGGGGGGGCGTGAGCGTATTCGGCAGATACGTTCTGAGGGAA<br>3′-CTGGTGGGAAAAACTATATTATATGAATATCGCCCCCCCGCACTCGCATAAGCCGTCTATGCAAGACTCCCTT	5	2
M11M17	5′-GACCACCCTTTTTGATATAATATACTTATAGGGGCGGGGCGTGAGCGATAAGCGCAGATACGTTCTGAGGGAA<br>3′-CTGGTGGGAAAAACTATATTATATGAATATCCCCGCCCCGCACTCGCTATTCGCGTCTATGCAAGACTCCCTT	6	2
M13M17	5′-GACCACCCTTTTTGATATAATATACTTATAGCGGGTGGGTGCGAGCGATAAGCGCAGATACGTTCTGAGGGAA<br>3′-CTGGTGGGAAAAACTATATTATATGAATATCGCCCACCCACGCTCGCTATTCGCGTCTATGCAAGACTCCCTT	6	3
M14M17	5′-GACCACCCTTTTTGATATAATATACTTATAGCGGGGGGGCGTGAGCGATAAGCGCAGATACGTTCTGAGGGAA<br>3′-CTGGTGGGAAAAACTATATTATATGAATATCGCCCCCCCGCACTCGCTATTCGCGTCTATGCAAGACTCCCTT	6	2
M15M17	5′-GACCACCCTTTTTGATATAATATACTTATAGCGAGGGGGTGCGAGCGATAAGCGCAGATACGTTCTGAGGGAA<br>3′-CTGGTGGGAAAAACTATATTATATGAATATCGCTCCCCCACGCTCGCTATTCGCGTCTATGCAAGACTCCCTT	6	4
M-1***	5′-GACCACCCTTTTTGATATAAAATACCTATATCAATGGCCTCCCACGCATAAGCGCAGATACGTTCTGAGGGAA<br>3′-CTGGTGGGAAAAACTATATTTTATGGATATAGTTACCGGAGGGTGCGTATTCGCGTCTATGCAAGACTCCCTT	S6	1
primer 1	5′-GCGTAAGTCTCGAGAACTAGTTCCGTAAGATGCTTTTCTGTGACT	-	-
primer 2	5′-GCGTAAGTGCGGCCGCTTCGTTCCACTGAGCGTCAGA	-	-
primer 3	5′-GACCGAGATAGGGTTGAGTG	-	-
primer 4	5′-TTTGTGATGCTCGTCAGGGG	-	-

***** Single mismatch DNA targets that contain 6 bp PAM terminal mismatch

** Double mismatch DNA targets that contain 6 bp PAM terminal mismatch

*** In previous studies of St-Cascade [3,4], the M-1 position was considered to be part of the protospacer such that the St-Cascade consensus PAM was AA. Although M-1 is part of the actual protospacer at the acquisition stage, this nucleotide does not participate in basepairing with the target DNA (also shown in Supplementary Fig. 6C). For reasons of consistency with other Type IE Cascade complexes [5,6], we now consider the M-1 position to be part of the new AAN consensus PAM.

Supplementary Table 4. Sequences of crRNAs present in Cascade complexes used in this study.

crRNA #	Sequences of crRNA
1	5′-GUGAUCCUAUACCUAUAUCAAUGGCCUCCCACGCAUAAGCGUUUUUCCCGCACACGCGGGG
2	5′-GUGAUCCUAGCGCGAUAUCAAUGCGCUCCCACGCAUAAGCGUUUUUCCCGCACACGCGGGG
3	5′-GUGAUCCUAUACUUAUAGCGCGUGCGUGCGAGCGAUAAGCGUUUUUCCCGCACACGCGGGG
4	5′-GUGAUCCUAUACUUAUAGCGAGCGCGUGCGAGCGAUAAGCGUUUUUCCCGCACACGCGGGG
5	5′-GUGAUCCUAUACUUAUAGCGAUGCGGUGCGAGCGAUAAGCGUUUUUCCCGCACACGCGGGG
6	5′-GUGAUCCUAGCGCGAUAUCAAUGCGCUCCCACGCAUAAGCGUUUUUCCCGCACACGCGGGG

Supplementary Table 5. Oligonucleotide sequences used for the fluorescence measurements. PAM for Cascade is coloured yellow, matching part of the target sequence – green, mismatched bases of the target sequence – red, flipped out bases – grey.

DNA substrate	Sequences of dsDNA	crRNA #
WT	5′-GGACCACGCATAATATACCTATATCAATGGCCTCCCACGCATAAGCAGTG-*[Cy3]*<br>3′-CCTGGTGCGTATTATATGGATATAGTTACCGGAGGGTGCGTATTCGTCAC-*[Cy5]*	1
M3	5′-GGACCACCCATAATAGGGCGATATCAATGCGCTCCCACGCATAAGCAGTG-*[Cy3]*<br>3′-CCTGGTGGGTATTATCCCGCTATAGTTACGCGAGGGTGCGTATTCGTCA-*[Cy5]*	6
M5	5′-GGACCACCCATAATAGCGGGATATCAATGCGCTCCCACGCATAAGCAGTG-*[Cy3]*<br>3′-CCTGGTGGGTATTATCGCCCTATAGTTACGCGAGGGTGCGTATTCGTCA-*[Cy5]*	6
M11	5′-GGACCACCCATAATATACTTATAGGGGCGGCGCGTGAGCGATAAGCAGTG-*[Cy3]*<br>3′-CCTGGTGGGTATTATATGAATATCCCCGCCGCGCACTCGCTATTCGTCAC-*[Cy5]*	2
M17	5′-GGACCACGCATAATATACCTATATCAATGGGCTCCCACGCATAAGCAGTG-*[Cy3]*<br>3′-CCTGGTGCGTATTATATGGATATAGTTACCCGAGGGTGCGTATTCGTCAC-*[Cy5]*	1
M21	5′-GGACCACGCATAATATACCTATATCAATGGCCTCGCACGCATAAGCAGTG-*[Cy3]*<br>3′-CCTGGTGCGTATTATATGGATATAGTTACCGGAGCGTGCGTATTCGTCAC-*[Cy5]*	1
M26	5′-GGACCACGCATAATATACCTATATCAATGGCCTCCCACGGATAAGCAGTG-*[Cy3]*<br>3′-CCTGGTGCGTATTATATGGATATAGTTACCGGAGGGTGCCTATTCGTCAC-*[Cy5]*	1
M32	5′-GGACCACGCATAATATACCTATATCAATGGCCTCCCACGCATAAGGAGTG-*[Cy3]*<br>3′-CCTGGTGCGTATTATATGGATATAGTTACCGGAGGGTGCGTATTCCTCAC-*[Cy5]*	1
Not matching	5′-GGACCACCCATAAGCTGTCTTTCGCTGCTGAGGGTGACGATCCCGCAGTG-*[Cy3]*<br>3′-CCTGGTGGGTATTCGACAGAAAGCGACGACTCCCACTGCTAGGGCGTCAC-*[Cy5]*	1
M27-32	5′-GGACCACGCATAATATACCTATATCAATGGCCTCCCACGCTATTCGAGTG-*[Cy3]*<br>3′-CCTGGTGCGTATTATATGGATATAGTTACCGGAGGGTGCGATAAGCTCAC-*[Cy5]*	1

Supplementary Table 6. Precise n values used to derive statistics.

Figure 2d											
Torque	8 bp	Torque	10 bp	Torque	12 bp	Torque	14 bp	Torque	16 bp	Torque	22
-6.291	91	-4.991	298	-4.319	139	-3.704	131	-3.437	430	-3.437	129
-6.477	43	-5.307	362	-4.663	194	-3.958	169	-3.704	391	-3.704	87
-6.66	100	-5.712	396	-4.991	233	-4.201	185	-3.958	348	-3.958	38
-6.841	98	-6.005	449	-5.307	263	-4.436	238	-4.201	260		
-7.019	102	-6.291	495	-5.712	233	-4.663	466	-4.436	264		
-7.195	109	-6.66	523	-6.005	228	-4.883	515	-4.663	186		
-7.369	87	-6.931	493	-6.291	508	-5.098	450				
-7.54	107	-7.195	373	-6.66	401						
-7.709	127										
-7.877	130										

Figure 2e							
Torque	10 bp	Torque	12 bp	Torque	14 bp	Torque	16 bp
-4.991	298	-4.319	139	-3.704	131	-3.437	430
-5.307	362	-4.663	194	-3.958	169	-3.704	391
-5.712	396	-4.991	233	-4.201	185	-3.958	348
-6.005	449	-5.307	263	-4.436	238	-4.201	260
-6.291	495	-5.712	233	-4.663	466	-4.436	264
-6.66	523	-6.005	228	-4.883	515	-4.663	186
-6.931	493	-6.291	508	-5.098	450		
-7.195	373	-6.66	401				

Figure 3d-e												
	C:C				C:T				C:A			
Torque	k_1	k_2	k_3	k_4	k_1	k_2	k_3	k_4	k_1	k_2	k_3	k_4
-6.101	13	13	1795	1795								
-5.908	26	26	2579	2579								
-5.712	16	16	1475	1475								
-5.512	22	22	2615	2615								
-5.307	31	31	2745	2745								
-5.098	38	38	2219	2219	27	27	6129	6129				
-4.883	37	37	1309	1309	18	18	6214	6214				
-4.663	99	99	1934	1934	94	94	14464	14464				
-4.436	146	146	748	748	109	109	8830	8830				
-4.201	195	195	331	331	221	221	9273	9273	53	53	335	335
-3.958	257	257	215	215	353	353	7753	7753	67	67	462	462
-3.704					265	265	2879	2879	108	108	575	575

Figure 4b-c											
Torque	WT	Torque	M5	Torque	M7	Torque	M11	Torque	M14	Torque	M17
-7.54	34	-7.54	50	-7.54	45	-6.66	37	-7.54	46	-7.54	44
-7.108	31	-7.108	46	-7.108	53	-5.712	49	-6.66	42	-7.108	28
-6.66	28	-6.66	42	-6.66	58	-5.203	74	-5.712	42	-6.66	29
-6.196	38	-6.196	57	-6.196	53	-4.663	60	-5.203	47	-6.196	24
-5.712	46	-5.712	38	-5.712	107	-4.378	60	-4.663	46	-5.712	29
-5.203	39	-5.203	52	-5.461	58	-4.08	52	-4.378	46	-5.203	41
-4.663	38			-5.203	60	-3.768	31	-4.08	54	-4.663	52
-4.08	28			-4.4	28			-3.768	48	-4.08	32
-3.437	35							-3.437	48	-3.768	41
-3.08	35									-3.437	39
										-3.08	44

Figure 6c		Figure 6d					
Distance	N	M11M17		M11		M17	
		Torque	N	Torque	N	Torque	N
11	76						
13	35	-5.712	75	-6.66	37	-7.108	28
14	41	-6.101	56	-5.712	49	-6.66	29
15	31	-6.477	76	-5.203	74	-6.196	24
		-6.66	76	-4.663	60	-5.712	29
		-6.841	55	-4.378	60	-5.203	41
		-7.019	28	-4.08	52	-4.663	52
						-4.08	32
						-3.768	41
						-3.437	39
						-3.08	44

Supplementary Figure 4g												
	0.1 nM				0.5 nM				2.5 nM			
Torque	k_1	k_2	k_3	k_4	k_1	k_2	k_3	k_4	k_1	k_2	k_3	k_4
-4.436					141	141	46	46				
-4.663	271	271	69	69	207	207	101	101	158	158	63	63
-4.883					122	122	126	126				
-5.098	274	274	161	161	177	177	196	196	129	129	112	112
-5.307					135	135	266	266				
-5.512	268	268	610	610	136	136	209	209	42	42	116	116
-5.712					64	64	184	184				

Supplementary Figure 5e												
	C:A and C:A adjusted				Simulated R-loop				Simulated tweezers experiment			
Torque	k_1	k_2	k_3	k_4	k_1	k_2	k_3	k_4	k_1	k_2	k_3	k_4
-3.704	108	108	575	575	17	17	2574	2574	16	16	546	546
-3.958	67	67	462	462	15	15	2483	2483	14	14	376	376
-4.201	53	53	335	335	13	13	2505	2505	13	13	520	520

Supplementary Figure 6c	
Torque	N
-7.54	32
-6.66	41
-5.712	29
-4.663	30
-4.08	29
-3.437	19

References

1. Zadeh, J. N. *et al.* NUPACK: Analysis and design of nucleic acid systems. *J. Comput. Chem.* **32**, (2011).
2. Jung, C. *et al.* Massively Parallel Biophysical Analysis of CRISPR-Cas Complexes on Next Generation Sequencing Chips. *Cell* **170**, (2017).
3. Sinkunas, T. *et al.* In vitro reconstitution of Cascade-mediated CRISPR immunity in Streptococcus thermophilus. *EMBO J.* **32**, 385–94 (2013).
4. Szczelkun, M. D. *et al.* Direct observation of R-loop formation by single RNA-guided Cas9 and Cascade effector complexes. *Proc. Natl. Acad. Sci. U. S. A.* **111**, (2014).
5. Mulepati, S., Héroux, A. & Bailey, S. Structural biology. Crystal structure of a CRISPR RNA-guided surveillance complex bound to a ssDNA target. *Science* **345**, (2014).
6. Hayes, R. P. *et al.* Structural basis for promiscuous PAM recognition in type I–E Cascade from E. coli. *Nature* 1–16 (2016). doi:10.1038/nature16995
